# Extra Supplement—The Nursing Mirror

**Published:** 1889-03-02

**Authors:** 


### The Nursing Mirror.

[*Extra Supplement*

All Contributions for this Supplement should be addressed "The Nursing Mirror," care of The Editor, 38, Old Jewry, E.C.

### En Passant.

THE YORK HOME.—This nursing institution has a staff of a sister-in-charge, 35 nurses, and eight probationers. The total receipts last year were £1,567; the nurses received as wages £82 6s. 9d. We leave these figures to speak for themselves. The report stated that the total number of cases nursed last year had been 627, as against 490 in 1887, being a total increase of 137 cases. The increase in the charitable work was represented by 114, whereas the increase in the cases nursed for payment had been 23. There had been during the year 40 cases of typhoid fever, 20 of scarlatina, 2 of diphtheria, and 2 of puerperal; 90 surgical cases, 26 mental cases, and 55 obstetrical cases.

AMERICAN NOTES.—"Nursing over here is exciting much interest at present, and we are all anxious to have Miss Clara Barton's report of how the yellow fever was nursed at Jacksonville. I sent you before a copy of "The Nightingale"; now I send you a copy of a new nursing paper edited by Miss M. E. Francis, formerly superintendent of Buffalo Hospital, called "The Trained Nurse." From it you will see that Miss E. Faining, who trained at Massachusetts, has been appointed matron of the Morton Hospital, built by Mrs. Kimball. The superioress of the German Hospital at Philadelphia is Wanda, Baroness Oertzen, and a woman who is not above her work in spite of her title. Bellevue is progressing, and as famous as a training school as ever. Mrs. Griffen, one of the presidents, was one of the four women attached to the hospital ship *Daniel Webster* during the civil war. Some day I hope to send you a longer account of her experience and further copies of our two nursing papers."

JEWISH NURSES.—The article in the *Jewish Chronicle* on this subject, which we noticed a fortnight since, has called forth much correspondence in the paper in which it appeared. Dr. Abraham Cohen writes an admirable letter, in which he points out that the London Hospital—the greatest hospital in the kingdom, and one of the best training schools for nurses—is quite unsectarian, and receives Jewesses as probationers. Then there are other letters of less import, for some people rush into print on every occasion, their only desire being to advertise themselves. For instance, what good could come of forming a committee? Why advocate increased red-tapism and fuss, unless the desire is to increase the sense of personal importance? There are the training schools, let the nurses enter; and let the Christians stand aside, and not force themselves forward in regard to a question which is purely Jewish, and difficult for outsiders to comprehend.

NURSING IN SOUTH LONDON.—An interesting meeting was held in St. Matthew's Hall, Lavender Hill, last Saturday, to hear the report of the last year's work of the South London District Nursing Association. The report was a good one, for the association received a legacy of £500 last year, and also £250 from the concert at Grosvenor House. On the strength of this generosity the Committee added two more nurses to their staff, and also undertook to train a lady probationer; there are therefore now eight inmates of the Home—Miss Rayner (the superintendent) six nurses, and one probationer. Of course, increased subscriptions are required to meet the increase of expenditure, and the Rev. Edward Venables, and others who addressed the meeting, pleaded for further support for the Association. After the meeting there was tea and talk, when Miss Rayner and the nurses very kindly answered all questions, and gave graphic particulars of some of their cases. The Nurses' Home, which adjoins the hall, was also open to inspection.

COMBINATION OF PRIVAVE NURSES.—The number of grumbling letters, which we are receiving at present from private nurses, is something enormous; but we do no see what good is to be got from grumbling. The spirit of fault-finding is an evil one; it has no advantages as long as it is confined to words. If things are so bad at some of the homes as our correspondents state, why do not all the nurses of the home sign a protest and send it in to the manager of the home? On the question of extra fees for mental and infectious cases the nurses ought certainly to combine throughout the length and breadth of the country to demand a portion of these fees. It is a very weak proceeding to send us grumbling letters which we are implored to print anonymously; until nurses have more courage and strength of character, of course those in power will not help them. We can print no more letters on this subject unless they contain practical suggestions, or unless we are allowed to print the writers' names. Surely the best plan would be for private nurses to hold a public meeting and discuss their grievances?

BIRMINGHAM NURSES.—The Midland Counties' Training Institution for Nurses held its annual meeting on February 22nd, when there was a large gathering of the friends of the institution. The Mayor presided, and made an excellent speech, as did also Canon Wilkinson. The report of the committee stated that nurses had attended 795 families during the year. There were on the staff eighty-three trained nurses and ten probationers. The committee acknowledged the valuable services rendered by Mrs. Diamond, the lady superintendent, and thanked the nurses for their endeavours to do faithfully the duties allotted to them. The financial record was again satisfactory, for whilst the subscription list amounted to only £199, the nurses' pension fund had risen to £1,295; and though this might appear a large sum, it was far below the necessities of the institution. The sinking fund for providing new premises at the end of the present lease amounted to £565. The committee directed attention to the fact that the whole of the subscription income of the institution was absorbed by the expenditure connected with the District Nursing Society. Why do not the committee join their pension fund to the National Pension Fund? Then the sum they have in hand would secure larger results.

THE INTERVIEWER AGAIN.—The new matron of Northampton Infirmary, whose appointment we noticed a fortnight ago, has been interviewed by the *Northampton Mercury* reporter, with the following result:—"Miss Winterton is a smart little woman just over 30, whose pleasant features seem to be the index of a nature kindly and sympathetic, yet firm and resolute.—'Yes, I have had a rather varied experience,' says the lady in answer to a question. 'I have had seven years of hospital work, some of it spent in England and some in Egypt during the war. My first hospital? Oh, St. Thomas's in London. I went there in 1881. After gaining experience there I thought I would go abroad. You will remember the Egyptian war broke out in 1882. In August of that year I went out with a corps of nurses, and was stationed at Alexandria and Ramleh.'—'You were not actually near the place of operations where the fighting was going on?'—'No, we had hospitals built, you know, and the sick and wounded used to be drafted down to us. Some of the cases, of course, were very terrible.'—'When did you leave Egypt?'—'I came home in November. In December, 1883, I went to the Royal Infirmary, Liverpool. I am glad to come to Northampton, because this is my own county, and my friends live here. Yes, I know I shall like it. I love hospital work.' There were 37 candidates for the post of superintendent. The lady next to her was Miss Dora Pressland. Miss Pressland is a nurse of great skill and experience. She was selected to nurse Princ Albert Victor during his illness. Miss Pressland has just obtained an appointment near London."

### Nurses on Their Travels.

A Letter from Madeira.

Amongst the many interesting letters we have received on the stewardess question is a very practical one from a nurse who has lately made the voyage to Madeira. Her remarks about stewardesses and about life abroad are so excellent we propose to give them in full; and we hope to be favoured with a second letter from the same source shortly. We are quite sure that many nurses are entering their names on the register of stewardesses who have not fully nor carefully considered what they are rashly proposing to do. We can only beg them to read the letters we are now publishing from nurses who have tried the experiment, and who acknowledge the many difficulties which mar this "life on the ocean wave." For instance, here is the letter from Tenchal, Madeira :—

"After the many letters of complaint published by nurses lately, perhaps a few lines from one having a good time may be acceptable. We came here in a Union boat; and remembering the proposal of nurse-stewardesses, I took particular note of their duties. Certainly they must be good sailors. Our second-class stewardess, a charming, motherly, but not elderly woman, said she was having a very easy passage, nevertheless I only saw her on deck once, except in port; and I would remind nurses that many who, like myself, enjoy a good toss in the open air, would find it very different going from one sick passenger to another, with only the stagnant air of the salon for change, and generally a fattish bilious passenger's baby in your arms betweenwhiles. There certainly is money in it, but I should advise a short trip first. At Lisbon we saw a sight which would have converted his Holiness himself to cremation. Imagine a long low marble chapel, round which it is the ghastly custom of the Portuguese Royal family to range their dead in coffins that look like over-ornamented travelling trunks All but two were fastened with very ordinary brass locks. These two were unlidded and covered with a sheet of glass. One held a dead baby, dreadful in colour, but with something soft and pretty about the little face and simple white frock. The other the guardian evidently considered it the chief attraction, and would by no means let us off mounting the three wooden steps which lead up to it, he striking matches the while that we might clearly see the old face, three years dead, within. It was the father of the present king, embalmed, I suppose, but with the flesh shrivelled, brown and green, and great patches of mildew spreading over the grand blue uniform. I have been nine years a nurse, but I never knew till then how hideous death is. Coming out we stumbled on a christening, and a young woman in a very smart Portuguese costume held me up a delicious, warm, and rather pretty baby to kiss. It did me good, but I still dream of those gruesome features and hands. Madeira —or, at least, the hotel we are in—seems a paradise for nurses. After English hotels, where one has to coax and cringe to get even into the still-room for poultices, to be welcomed into the kitchen to make riveltia, even at the supreme moment when dinner is being dished up, is wonderful; and when the English proprietor and his w.fe kindly invite me to make myself at home everywhere and take what I wanted, it made me feel I was indeed abroad. Now for a few professional mems. Being all hills, there are no wheeled conveyances, but the four-post bedsteads, hung with chintz, mounted on narrow sledges, and drawn by oxen, do not shake the patients quite to jelly, as I expected. Marrow and watercresses are ordered here instead of codliver oil. It may be an old substitute, but it is new to me, and were I a patient I should hail it with joy. We were warned of diarrhœa, but the fruit, though abundant, is not very seductive. Custard apples and loquats are the only new kinds I have seen. The former, a handsome green fruit, very like a half-blown artichoke, inside filled with a pear-like substance, and large black beans; it does not come up to its name. Loquats are like small yellow plums, sour and unwholesome looking. Of course there are heaps of oranges, bananas, and good apples. Strawberries are just coming in. The climate is heavenly, warm, even, and breezy. Yesterday was a festa. There was great tinkling of a queer fiddle-shaped instrument, name unknown, and playing by men in the road of a sort of primitive croquet without hoops, but the course, requeting, and tight croquet much as we had it some fifteen years ago."

The above is a truly interesting and intelligent account of a "Nurse on Her Travels," and we quite agree with the writer that the life of a stewardess is not all lemonade and cheese-cakes, and that only those who have a natural aptitude for overcoming difficulties, and are good sailors or great experience, should offer themselves for these posts.

### Starting a District Nurse.

By One Who Has Done It.

When we decided in our small town of Sanstead that a parish nurse was a necessity, we first took counsel with our rector and parish doctor, and on their recommendation formed a committee of twelve of the most important inhabitants of the town. We had six ladies on the committee and six gentlemen, and one of the ladies, who had trained at St. Mary's Hospital, was appointed secretary and lady superintendent. At the second meeting of the committee our secretary came armed with reports from the East London Nursing Society, of 49, Philpot Street, Stepney, the National and Metropolitan Nursing Association, of Bloomsbury Square, and others; and from facts found in these reports she stated that £70 a-year was needed to keep a nurse; and that for the first year £100 would be needed to provide nursing appliances and stores, and furniture for the nurse's room. We then and there drew up an appeal for funds, of which we determined to have 500 copies printed, and then we divided the town into twelve districts, and each undertook to canvass a certain district. The next meeting of the committee took place a month later, when we were able to announce that the necessary funds were collected, and that now we had to find the nurse. We advertised in The Hospital and received numerous replies, from which the three most promising were selected and laid before the committee. One, Nurse Evans, who had trained at the London Hospital and Queen Charlotte's, and been parish nurse in a small town before, was interviewed by the secretary. The nurse proved to be an energetic, kindly woman of 35, and she was forthwith engaged to come to us when her month's notice was up. We secured two comfortable rooms over a small baker's shop, in the poorest part of the town, and then we drew up the following rules for the guidance of the nurse :—

(1) A nurse shall be engaged by the committee at a salary of £50, two rooms being provided for her. (2) Nurse will be required to wear a uniform dress when on duty. (3) Nurse to receive one month's notice to quit, and to give the same. (4) Nurse is required to work eight hours daily, this time to be extended only under exceptional circumstances. (5) Nurse shall have eight hours for sleep, and at least two hours leisure daily. (6) Nurse will, as a rule, be employed on night duty only under exceptional circumstances. (7) Nurse is not allowed without the permission of the secretary, to give money or relief of any kind. (8) The nurse's work shall be entirely under the direction of medical men. (9) Nurse shall be responsible for the personal cleanliness of each patient under her charge, and for the care and cleanliness of the room. Nurse shall on every visit see that the rooms, furniture, and utensils of each patient are clean, or clean them herself. (10) Nurse shall not attend infectious cases; but when possible shall attend confinement cases, charging 5s. for every such case. (11) Nurse shall have two weeks' holiday in the year, and an evening off duty twice a month on application to the secretary. (12) Nurse shall visit the secretary every Monday morning, and give a full account of the past week's work, which shall be taken down in a written report, and laid monthly before the committee.

These twelve rules we found to answer admirably as a basis from which to work. At first the nurse had little to do, but when the people got to know her, her services were in great demand. At the request of the doctors, clergy, and committee she visited many sick, and soon no one of our poorer inhabitants fell ill without immediately sending for nurse. We lately held our first annual meeting, and all agreed that the report submitted was in every way satisfactory.

### Notes from Australia.

*(By Our Own Correspondent.)*

Melbourne, January 1st.

One large item of news is stirring the hospital world here just now—the fire at the Alfred Hospital on December 26th. It is supposed to have originated through some flaw in a flue. It was first noticed by some of the attendants, who at once raised the alarm. Special hose are supplied all over the building, and one of these was soon affixed to the stand-pipe, but when the tap was turned on the water merely dribbled out, and all hopes of extinguishing the fire in this way had to be abandoned. As soon as the first alarm had been given, Mr. J. F. Anderson, the secretary, telephoned for the brigades. They ran their hose out, only to find what the hospital employés had already discovered, that the pressure of water was most inadequate, and would hardly trickle through the branches. As the fire increased in spite of all efforts to subdue it, the occupants of the smaller wards were removed down the large stone staircase. The doors of the large ward upstairs were kept closed, and the secretary and the nurses were moving about amongst the patients encouraging them to remain quiet and not be alarmed, as the fire would soon be out. The conduct of the nurses is spoken of as admirable. The steam fire-engine had been got to work by this time, but the flames had obtained such a hold on the roof of the building, and there was such a strong wind blowing, that it soon became evident the whole roof must go. Then came the terrible work of removing the patients. There was no undue excitement. Everything was done systematically; but the work had not been undertaken too soon, for the last patient had barely been removed from the ward when the roof fell in. Beds were provided in the operating and out-patients' room, and this, with a little judicious management in the placing of the beds in the other wards and the abandonment of the private rooms by the resident medical staff, enabled all the patients to be got under cover. The day was fortunately beautifully mild, and none of the patients have suffered either from the excitement or the exposure. The damage is estimated at £2,500, and a rebuilding fund is already being largely subscribed for.

I hear that Dr. Louis Henry will represent the *British Medical Journal* at the Colonial Medical Congress, which meets here next week, and "your own correspondent" will of course send reports to The Hospital.

The quarterly report of the executive committee of the Charity Organisation Society, just issued, stated that during the last three months the applications of 73 persons for charitable relief had been dealt with. Of these cases 44 were relieved and four were assisted. Of the 73 applications inquired into 27 were hospital cases. From September 1st to the 30th ult. only eight new members had joined the society, notwithstanding that some seven hundred circulars had been sent out. The receipts for the three months were £91 5s. There was now a balance in hand of £119 18s. 8d. No less than forty overdue subscriptions remained unpaid.

The District Nursing Society also reports a balance in hand of £334. This must astonish you in England, who love to have your charities in debt. The district nurses visited 36 cases during November, and their work is appreciated.

There have been two independent movements of late to bring desirable immigrants into this colony. One is that of the Messrs. Chaffey to get out a population for their irrigation colony of Mildura; the other, that of certain citizens of Melbourne to supply the want, painfully felt and loudly complained about, of young women suitable for and disposed to domestic service. The first plan is flourishing, but in the absence of a Mrs. Fry, the second scheme is in a merely tentative stage.

Typhoid fever is still very bad here, and no wonder, since all the drainage goes into the lagoon, which is full of decaying matter, and a most glorious field for the cultivation of all those microbes about which Mr. Pearson has lately been lecturing us. There was a scheme for fresh drainage works, but the Bill has been rejected by the Legislative Council, owing to some difficulty about "who is to pay." This is ridiculous in a rich town like Melbourne.

During the month of November, the births registered in Melbourne exceeded the deaths by 510, or 62 per cent. Six deaths were due to homicide, and nine to suicide.

The Ladies' Sanitary Association of Sydney is doing good work; ladies take a much more prominent part in health matters in the Colonies than they do in England.

*(To be continued.)*

### Everybody's Opinion.

*[Correspondence on all subjects is invited. No communications can be entertained if the name and address of the correspondent is not given, or unless one side of the paper only be written on.]*

### CERTIFICATES.

A. E. S., Matron, writes:—A question which occupies a good deal of attention is the giving of certificates, and to my mind it is a very difficult one—a certificate means so much—on the other hand, so little. At the end of two years, when a probationer leaves, the certificate is thus worded —"A. B. finished her term of probation, took duty in male and female wards, also was on night and special duty, attended medical, surgical, and nursing lectures." Now, for all that she man be unfit to be a nurse; there may have been nothing really wrong in her conduct, and in a few years she may pull herself together, for one must always remember girls do much wrong from pure thoughtlessness. All this cannot be put on a certificate paper; and, again, if one refuses to sign a certificate, one takes the bread out of a woman's mouth. It is most difficult to act fairly to oneself and to the would-be nurse. The public will have certificates, so that we matrons must make the fact well understood that we will sign for no one who has not given real satisfaction. "Old heads can't be put on too young shoulders;" but one must not give certificates on too easy terms.

### THE COMBINATION OF PRIVATE NURSES.

Nurse S. writes: I have read "A Superintendent's" letter on "A Favourite Grievance" with some amusement, and much wonder that more of the same kind have not been written, and that superintendents have not been able to find better arguments with which to defend themselves. Everyone knows that it requires money to keep up institutions. It would require that to supply the ladies who start them with comfortable homes, gas, fire, food, etc. The nursing staff does not, however, derive much benefit from all that is spent on them. It is true they are supplied with work, but were there no institutions they would get work as well. What they complain of is that institutions by monopolizing all the work that is to be had force them to join them, and to join too at a disadvantage, or starve. I think this form of reasoning will explain our friend's suggestion about working for a penny a day. Probably she would give no more if she could help it. It is absurd to talk of free-will when one is forced by circumstances. There is no free-will about the matter at all; nurses must take what they can get. It is a widespread as well as a favourite grievance—this constant complaint about insufficient pay. We have it from all classes of the employed, and it seems to be impossible to obtain fairness or honesty from employers except by force. Now the only force employés have at their command is refusal to work unless properly remunerated—a state of affairs which means a good deal of suffering to them until their demands are satisfied. Even this poor weapon is not at the disposal of private nurses. They cannot all strike at once; they are scattered all over the town or country, and common humanity forbids them to leave their cases at a moment's notice. They can only, as they return to their institutions, refuse to work, one by one, and get turned out for their pains, whilst their places are easily filled. As to saying that the nurses cost their superintendents a considerable sum, it is rubbish. Many a private nurse only spends a couple of weeks in scattered intervals out of the whole year at the Home. If she is sick, she is sent to a hospital to be supported by charity. She earns on an average £60 a-year, out of which she gets £20 or £25, paying her superior £40 for two weeks' accommodation and a few doubtful advantages. Now nurses are not stupid; they are quite content to give a fair return for all that they receive—but, for heaven's sake, let it be a fair one! I say nothing about the practice of charging extra fees for fever cases, etc., from which the woman who runs all the risk is given nothing. That fact is so glaringly dishonest that it needs no comment.

NURSES AS STEWARDESSES.

Miss Harvey writes from Wisconsin, U.S.:—Can you give me information regarding the nurse or stewardess? I saw a notice in The Hospital. Being a good sailor, and very fond of the sea, I think I should like to get a post on a vessel. May I trouble you for a reply? Regret I am unable to send you a stamped envelope. I have not an English stamp, and no means of procuring one. I think of returning home to England shortly. A friend sends me The Hospital each week, and I look forward to receiving it with great pleasure,

PRIVATE NURSES.

Nurse M. M. writes:—I am very pleased to read Nurse Rose's defence of the Home to which we both belong, and heartily agree with all she says. I have belonged to the institution ever since it was opened, and have always found the Sisters most kind and considerate to all nurses who did their duty. The nurses who complain have themselves to thank for the few rules that have been recently made, which really do not affect a conscientious nurse, who would never think of doing the things they forbid. I feel sure that several other of our nurses quite agree with Nurse Rose and myself.

### The Nurses' Bookshelf.

[Books for Review should be sent to The Editor, The Lodge, Porchester Square, W.]

### A Handbook for Women.*

This well-known handbook and directory is published in three parts this year, as well as in the ordinary shilling volume. The separate parts are sold for 6d. each. Part I. deals with social, religious, and benevolent institutions for women. Part II. with educational, literary, and technical pursuits; and Part III. with the subjects of medicine, midwifery, and nursing. All the particulars of the newest convalescent homes, or societies for aiding women are to be found here, and the articles on such subjects as nursing and education are eminently practical. We can only suggest one improvement to this useful book, and that is a fuller index compiled with a little more care. The index is always a very important part of a directory, and is worth more thought and consideration than it often gets.

### Appointments.

[It is requested that successful candidates will send a copy of their applications and testimonials, with date of election, to The Editor, The Lodge, Porchester Square, W.]

Darenth Asylum.—Mrs. Merina Williams has been appointed as matron of the above asylum at a salary of £100 per annum, with board, furnished apartments, and washing. Mrs. Williams is a widow, is 39 years of age, and holds at the present time the appointment of matron of the Junior Girls', Infants', and Convalescents' School, of the South Metropolitan School District, Sutton, Surrey, a post to which she was appointed two and a-half years ago. Previously to her appointment at Sutton, Mrs. Williams was for 18 months matron of the Royal Albert Asylum at Lancaster; for two years matron of the Peckham House Asylum; and for three years assistant matron at the Earlswood Asylum, Surrey.

Charing Cross Hospital.—Amongst the new sisters who will commence work at this hospital, under Miss Gordon, are Miss K. Peter, late of Jenny Lind Infirmary; Miss Hawdon, of Stafford Infirmary; Miss Dunbar, Miss Ransford, and Miss Portus.

### Lectures.

On March 8th, at the Midwives' Institute, 15, Buckingham Street, Strand, at a quarter to eight, Dr. A. Jamison on "The Pulse and Its Teachings." Admission to members' friends, 6d.

On March 14th, at the Sanitary Institute, 74a, Margaret Street, at Five p.m., Professor George Milne Murray, on "Fungi in Relation to Putrefaction and Sanitation. Admission 6d.

* "The Englishwoman's Year Book." By L. M. H. (Hatchards. Price 1s.)

### Presentations.

On February 13th, at Preston and County of Lancaster Royal Infirmary, upon Miss Pigott completing seven years of office as matron-superintendent, a handsome solid silver spoon warmer was presented to her by the nurses of that institution trained under her, as a mark of their esteem and goodwill. The head nurses provided a *récherché* tea for the staff, after the full appreciation of which all enjoyed watching the farce of "Turn Him Out," performed by the house-surgeons and head-nurses, and listening to a general entertainment. A dance for the nurses brought a very pleasant evening to a close. The performance was repeated a few nights after in one of the large wards for the patients.

### Vacancies.

The following vacancies are announced:—

**Matrons.**

Weymouth Royal Hospital, £35.
Holborn Union Infirmary, £35.
Hospital for Incurables, Manchester; £60.

**Head Nurse.**

St. Cuthbert's Hospital, Edinburgh; £40.

**Nurses.**

Bromley Sick Asylum (night), Two; £21.

Eastbourne Nursing Institution.

Glasgow Sick Poor Association.

London Temperance Hospital (night).

Nursing Institution, 96, 97, 98*a*, Wimpole Street, London, W., Mr. Wilson has several vacancies for thoroughly-trained Nurses.

Eston Hospital; £25.

Preston Infirmary; £20.

Portsmouth Royal Hospital; £25.

Hampstead Home Hospital.

London Temperance Hospital, Hampstead Road; £25.

Southport Infirmary; £20.

Manchester Hospital for Consumption, Bowdon, Cheshire.

Worcester City and County Nursing Institution; several.

St. John's Nurses' Institution, Southport; several.

West Bromwich District Hospital; £22.

Kingston Home, Hull.

St. John's Institution, Southport.

United Hospital, Bath; £26.

Colchester Hospital; £24.

Tewkesbury Hospital (night); £20.

South Devon Hospital; £25.

Bedford Institute; £25.

Ipswich Hospital (night); £20.

Brockley Nursing Home.

Nottingham Institution.

Nurses' Home, Truro.

West Bromwich District Hospital; £22.

West Norwood Nursing Institution; several.

Hereford General Infirmary; £20.

Rous Hospital, Newmarket; £20.

Southport Trained Nurses' Home; several.

Bradford Infirmary; £25.

**District Nurses.**

Holloway Road Home.

Bolton.

Stone Nursing Association; £52.

**Midwives.**

Glasgow Sick Poor Association.
Balmain, Highgate Hill.

**Probationers.**

Whitechapel Infirmary.

Durham County Hospital.

Batley Cottage Hospital, Yorks.

Hammerwich Cottage Hospital.

### Notes and Queries.

To Correspondents.—1. Questions may be written on post-cards. 2. Advertisements in disguise are inadmissible. 3. In answering a query please quote the number. 4. If a private answer is desired, a stamped addressed envelope must be forwarded.

**Queries.**

(116) *Spinal Carriage.*—Has anyone a second-hand good spinal carriage to sell cheap to a great sufferer without means?—*Miss L. Lees, 102, Lancaster Gate, W.*

(118) *Experience.*—Will any one kindly inform a trained nurse of any large hospital where she could be received for twelve months to gain further experience?—*Norwood.*

(119) I shall feel obliged if you can inform me through The Hospital if Sunlight Soap is at all injurious when used for the skin?—*A.*

(120) *Medical Dictionary.*—Can you tell me of a less expensive dictionary than advertised in last week's Hospital suitable for a nurse?—*A Private Nurse.*

**Answers.**

(110) *Midwifery Training.*—Apply to St. Mary's Mission House, Plaistow; or training can be had from the Glasgow Maternity Hospital for £10.

(111) *Register for Monthly Nurses.*—I believe there is such a register at the Midwives' Institute, 15, Buckingham Street, Strand.—*Nurse Nina.*

(115) *Complaints from Nurses.*—We see no reason why we should insert L. Taylor's letter, unless she sends proofs of the charges she brings forward.

(117) *Sister Clark.*—Your letter has been forwarded to the authorities of the Fund.—*Editor.*

(121) *Margaret.*—The address of the Liverpool Lint Company is Mark Street Mill, Netherfield Road, Liverpool.

(122) *Trained Hospital Nurse.*—Quain's "Dictionary of Medicine" may be had of the Publisher of The Hospital at 27s. 6d., carriage free.

